# Nationwide guideline implementation: a qualitative study of barriers and facilitators from the perspective of guideline organizations

**DOI:** 10.1186/s12913-025-12270-2

**Published:** 2025-01-27

**Authors:** Andrea C. Thoonsen, Anika Gans, Toby T. Broeders, Ilse van Beusekom, Diana M. J. Delnoij, Martine C. de Bruijne, Hanneke Merten

**Affiliations:** 1 Amsterdam UMC, Department of Public and Occupational Health, Vrije Universiteit Amsterdam, Amsterdam Public Health research institute, Van der Boechorststraat 7, NL-1081 BT Amsterdam, The Netherlands; 2 Zorginstituut Nederland, Department of Care, Willem Dudokhof 1, NL-1112 ZA Diemen, The Netherlands; 3 Erasmus Universiteit Rotterdam, Erasmus School of Health Policy & Management, Health Care Governance, Burgemeester Oudlaan 50, Rotterdam, The Netherlands

**Keywords:** Clinical practice guidelines, Guideline adherence, Implementation science, Guideline organizations, Quality improvement, Barriers and facilitators

## Abstract

**Background:**

Although the number of Dutch guidelines is growing, their uptake and impact in clinical practice lag behind. Dutch guideline organizations, including guideline developers, governmental agencies, health insurers and other national organizations, play a crucial role in developing, authorizing and/or supporting the use of guidelines. They influence end users' awareness, accessibility, understanding, acceptability and applicability of guidelines. In this study, we explored the barriers and facilitators that representatives of guideline organizations perceive in nationwide guideline implementation.

**Methods:**

In this qualitative study, we conducted semi-structured interviews with 35 representatives of 24 different guideline organizations. We employed framework analysis, using the updated Consolidated Framework for Implementation Research (CFIR), and thematic analysis to guide our data analysis and synthesis.

**Results:**

We found 45 different implementation barriers and 35 implementation facilitators. We identified seven overarching themes of interrelated barriers and facilitators that extended across the stakeholders involved and domains within the updated CFIR. These included 1) healthcare demand and resource availability, 2) implementation knowledge and expertise, 3) guideline characteristics: representation, evidence base and design, 4) partnerships and collaboration, 5) characteristics of guideline implementation planning, execution and evaluation strategies, 6) characteristics of healthcare professionals: need, capability, opportunity and motivation, and 7) legal and regulatory compliance.

**Conclusions:**

We obtained valuable insights into the complex dynamics of barriers and facilitators perceived by guideline organizations in nationwide guideline implementation. Our findings help explain why healthcare professionals and healthcare facilities may (not), slowly or inconsistently adhere to guideline recommendations in practice. The identified barriers and facilitators provide guidance for policymakers to re-evaluate and improve nationwide quality and guideline implementation policies, to eventually improve clinical practice and health outcomes for patients.

**Supplementary Information:**

The online version contains supplementary material available at 10.1186/s12913-025-12270-2.

## Background

Clinical practice guidelines are important means of optimizing patient care. Guidelines include recommendations informed by systematic reviews of the current scientific evidence and assessments of the benefits and harms of alternative care options [1]. They support healthcare professionals and patients in making informed medical decisions by promoting interventions of proven benefit (appropriate care) and discouraging ineffective or potentially harmful ones (low-value care) [2, 3]. Beyond guiding clinical practice, guidelines also support medical education, highlight important research gaps, and facilitate the monitoring and evaluation of care quality and cost-effectiveness [4]. In countries facing growing demand for healthcare, rising costs, healthcare resource shortages, and the environmental impact of healthcare practices, guidelines can play a crucial role in healthcare regulation [3, 5, 6]. As a source of readily available evidence, guidelines have shown the potential to improve the process of care and patient outcomes, support appropriate care delivery and reduce treatment inequality [3, 6, 7].

The Netherlands is a pioneer in the development of guidelines. Since their initiation in 1982, Dutch guidelines have become the cornerstone of quality policy in prevention, diagnosis, treatment, and healthcare organization [8]. As of 2024, there were approximately 2,200 guidelines for medical specialists, 350 for nurses, 140 for general practitioners, and 50 for mental health professionals [9–12].

The value of guidelines depends not only on the quality of their recommendations, but also on their application in clinical practice [13, 14]. While deviations based on valid medical reasons and considering the specific circumstances of individual patients are permitted, Dutch law expects healthcare professionals and healthcare facilities to follow guidelines [15]. Hence, the primary responsibility for implementing these guidelines and reshaping clinical practice and organizational structures rests with these guideline end users.

Alongside healthcare professionals and facilities, another key group of stakeholders is fundamental to guideline development and plays an important role in their implementation: guideline organizations. This term encompasses a diverse group of centrally operating national organizations that, as part of their tasks, develop guidelines, authorize them and/or support their use in clinical practice. In the Netherlands, these include scientific and professional organizations, patient organizations, governmental agencies, health insurers and other national (umbrella) organizations. Examples are the Dutch College of General Practitioners (NHG) and the Knowledge Institute of the Dutch Association of Medical Specialists (KIMS). They influence factors such as end users' awareness, accessibility, understanding, acceptability and applicability of guidelines [16–18]. Furthermore, they can actively stimulate local implementation by selecting, tailoring and applying dissemination and implementation strategies [13, 18–21].

Although the number of Dutch guidelines is growing, research shows that their implementation in local policy and practice can be unpredictable, slow and complex, potentially resulting in suboptimal patient care and wasted resources [22–27]. It is important to recognize that just developing and publishing guidelines and expecting healthcare professionals and facilities to implement them does not guarantee uptake or improvements in practice [28].

Various determinants can influence the implementation of guidelines. These determinants can occur at the micro, meso and macro level, including those of the guideline, healthcare professional, healthcare facility, guideline organization and external environment [29]. Identifying the determinants, that is, the barriers and facilitators, is an important first step in enabling effective selection and tailoring of strategies to improve guideline implementation [13, 19–21].

Barriers and facilitators to implementation experienced at the healthcare professional or facility level have been extensively studied nationally and internationally [30–35]. In contrast, influential determinants perceived by (Dutch) guideline organizations have received less attention [16, 36]. By examining the barriers and facilitators encountered at their level, we may uncover structural issues, nationwide trends, or new influential determinants. Furthermore, it enables the creation of well-informed national policies and activities regarding the development, implementation, and evaluation of guidelines. Therefore, the aim of this study was to explore the barriers and facilitators that representatives of guideline organizations perceive in the implementation of guidelines.

## Methods

We used the consolidated criteria for reporting qualitative research (COREQ) for reporting the methods and findings (see Supplementary Material 1) [37].

### Study design

A qualitative design with semi-structured interviews was used to gain in-depth insight into the implementation barriers and facilitators perceived by representatives of guideline organizations.

The current study is part of a larger qualitative interview project that also investigates the role of Dutch guideline organizations in the implementation process, the specific implementation planning approaches they utilize, the implementation strategies they employ, and how they monitor and evaluate guideline implementation. Findings related to these aspects are reported elsewhere [38].

The Medical Ethics Review Committee of Amsterdam University Medical Centers approved the study protocol. They concluded that this study did not fall within the scope of the Dutch Medical Research Involving Human Subjects Act, as representatives were not subjected to medical procedures or specific behavioral rules [39]. Therefore, no additional formal ethical approval was required (statement ID 2021–0417).

### Participants and sampling

The study population consisted of representatives of Dutch guideline organizations. We used purposive sampling methods to recruit a broad sample of these representatives [40]. Eligible guideline organizations were scientific/professional organizations, knowledge institutes, governmental agencies, health insurers, patient organizations and other national (umbrella) organizations that developed guidelines, published them and/or actively supported their use in clinical practice. Representatives were recruited based on their understanding of their organization's role or their own direct involvement in guideline implementation. We recruited potential participants through contact information obtained from guideline organization websites, contacts of the research team, as well as snowball sampling, where interviewed representatives recommended others [40]. Representatives were contacted via email or telephone. Prior to participation, they received an information letter containing study background, privacy information and informed consent information.

### Data collection

Interviews, conducted between March and October 2023, were held via videoconference or in person, depending on representatives’ preferences. Interviews were conducted by one or two researchers (TB, AG, AT), each with a background in health policy studies, completed training in interviewing techniques and prior interview experience. We employed a semi-structured approach using an interview guide (Supplementary Material 2) with primarily open-ended questions, while also allowing for a more in-depth exploration of emerging topics. The guide included questions about perceived implementation barriers and facilitators. As mentioned under ‘Study design’, this study was part of a larger qualitative interview project. Therefore, the interview guide also covered questions related to implementation planning, execution, monitoring and evaluation activities as well as general inquiries, such as opportunities for improvement.

At the beginning of each interview, representatives were asked for permission to record the session for transcription purposes. We obtained written and verbal informed consent from each representative. Interviews lasted between 30 and 100 min, and representatives received an interview summary for member checking afterward. All interviews were audio recorded and transcribed verbatim. Data collection continued until no new themes emerged, indicating data saturation.

### Theoretical framework, data analysis and synthesis

Interview transcripts were analyzed using the principles of framework analysis [41]. The updated Consolidated Framework for Implementation Research (CFIR) [29] guided our analysis. CFIR is a widely used framework for characterizing and classifying implementation determinants of healthcare innovations [42]. The 2022 CFIR update incorporates various recognized implementation theories, such as the COM-B constructs of the Behavior Change Wheel [43]. It organizes barriers and facilitators into five domains: 1) innovation (the guideline being implemented), 2) inner setting (the setting in which the guideline is implemented, e.g., healthcare facility), 3) outer setting (the environment in which the inner setting exists, e.g., the health system), 4) individuals (the roles and characteristics of individuals, e.g., healthcare professionals and guideline committee members) and 5) implementation process (the activities and strategies used for guideline implementation) [29].

We applied deductive coding to the data. We developed an initial codebook based on the implementation domains and determinants from the updated CFIR to categorize the barriers and facilitators that representatives identified. Furthermore, open coding was used to capture interesting aspects that emerged from the data, such as additional barriers and facilitators. The codebook was updated iteratively throughout the process. Two researchers (AT and either TB or AG) independently coded the first eight interviews to align their coding. Subsequently, one researcher (TB, AG, or AT) coded the remaining interviews, cross-checked by a second researcher (AT or HM). The final coding tree is provided in Supplementary Material 3. Coding was conducted using MAXQDA (version 2022).

After systematically categorizing the identified barriers and facilitators using the updated CFIR and organizing them in a data extraction template (Excel), we conducted a further analysis to explore their interactions and dynamics. This involved examining patterns, connections and influences between barriers and facilitators, as well as across different stakeholders and CFIR domains. Through this additional thematic analysis, we identified seven themes of barriers and facilitators, extending beyond the original CFIR domains. To provide a clearer understanding of the interactions and dynamics between determinants, the results are presented according to these themes.

## Results

### Participant characteristics

We interviewed 35 representatives of 24 different guideline organizations. An overview of the representatives and their respective guideline organizations is presented in Table 1. To ensure that the quotes could not be traced back to specific representatives, we grouped them using four overarching labels: guideline developers (scientific/professional organizations and knowledge institutes), governmental agencies, health insurers, and other national organizations. The findings are presented using these group labels. Eight representatives held dual roles, such as working at both a governmental agency and a professional organization. Additionally, four representatives actively served as healthcare professionals while employed at a scientific organization, and six representatives had prior experience working as healthcare professionals.

**Table 1:** Participant characteristics

Categories of guideline organizations	Guideline organizations	Number of representatives
Guideline developers (scientific/professional organizations and knowledge institutes)	Dutch Quality Alliance in Mental Health Care (Akwa GGZ)	1
	Netherlands Comprehensive Cancer Organization (IKNL)	1
	Knowledge Institute of the Dutch Association of Medical Specialists (KIMS)	2
	Dutch College of General Practitioners (NHG)	1
	Dutch Society for Internal Medicine (NIV)	2
	Dutch Society of Physicians for Respiratory Diseases and Tuberculosis (NVALT)	1
	Dutch Geriatrics Society (NVKG)	1
	Dutch Society of Obstetrics and Gynecology (NVOG)	2
	Netherlands Society of Cardiology (NVVC)	1
	Dutch Society for Surgery (NVvH)	1
	Netherlands Association of Nurses and Care Workers (V&VN)	2
	Netherlands Association of Sports Medicine (VSG)	2
Governmental agencies	Health and Youth Care Inspectorate (IGJ)	1
	Dutch Healthcare Authority (NZa)	1
	Ministry of Health, Welfare and Sport (VWS)	2
	National Health Care Institute (ZIN)	4
	Netherlands Organization for Health Research and Development (ZonMw)	1
Health insurers	Health insurance company	1
	Health Insurers Netherlands (ZN)	1
Other national organizations	Netherlands Federation of University Medical Centers (NFU)	1
	Dutch Hospital Association (NVZ)	1
	Netherlands Patients Federation (PFN)	1
	Health Care Evaluation and Appropriate Use (ZE&GG)	2
	Independent Clinics Netherlands (ZKN)	2

### Implementation barriers and facilitators

Representatives mentioned 45 different implementation barriers and 35 different implementation facilitators. Supplementary Material 4 provides a comprehensive list of the barriers and facilitators, classified according to the updated CFIR framework and illustrated by quotes.

We identified seven recurring themes of barriers and facilitators that were interconnected and extended across the five domains of the updated CFIR:

1. Healthcare demand and resource availability,
2. Implementation knowledge and expertise,
3. Guideline characteristics: representation, evidence base and design,
4. Partnerships and collaboration,
5. Characteristics of guideline implementation planning, execution and evaluation strategies,
6. Characteristics of healthcare professionals: need, capability, opportunity and motivation,
7. Legal and regulatory compliance.

We have outlined the barriers and facilitators according to these seven themes in the findings below and in Table 2.

**Table 2:** Implementation barriers and facilitators identified by guideline organization representatives, categorized using the updated CFIR and organized into seven overarching themes

CFIR categories of implementation determinants	Barriers	Facilitators
**THEME 1: Healthcare demand and resource availability**		
**Outer setting domain**		
Local attitudes	*No barrier identified in this category*	Increased implementation momentum/urgency/more attention to implementation
Local conditions	Due to the increasing demand for care and limited care provision resources, concessions must be made regarding quality, accessibility, and affordability, which means that not all guideline recommendations can be implemented	Due to the increasing demand for care and limited care provision resources, more attention is given to implementing appropriate care described in guidelines
Financing	Many guideline organizations lack standard budgets for implementation and instead depend on supplementary external funding	Various funders of guidelines require guideline developers to meet implementation criteria as a prerequisite for funding the development of guidelines
	The connection between quality of care and care reimbursement/financial incentives is weak and challenging to establish in the current healthcare system	Healthcare facilities receive financial compensation in exchange for creating an implementation action plan
	Fear of reduced revenue due to the de-implementation or relocation of certain care practices as recommended in the guideline	
	Implementation projects/programs and their funding are temporary and lack structural updates, leading to unsustainable implementation or continued use of outdated guidelines/implementation tools	
External pressure	Priority towards developing/revising guidelines rather than disseminating/implementing them	*No facilitator identified in this category*
*Available capacity / resources*^a^	Guideline organizations lack the capacity/resources to handle the multitude of (quality) tasks, including implementation of guidelines, and therefore have to determine which quality initiatives to prioritize	*No facilitator identified in this category*
**Inner setting domain**		
Relative priority	Healthcare facilities need to determine which quality initiatives (e.g. programs, guidelines) to prioritize and implement, given the abundance of initiatives, limited capacity/funding and the compatibility of the requested organizational changes	*No facilitator identified in this category*
Available resources	*No barrier identified in this category*	Healthcare facility has allocated funding to enable healthcare professionals to dedicate time to implementation
**Individuals domain (roles subdomain / characteristics subdomain)**^b^		
Implementation team members / opportunity	Guideline committee has insufficient capacity/time to plan/execute implementation strategies	*No facilitator identified in this category*
Implementation team members / motivation	Guideline committee lacks motivation to plan/execute implementation strategies	*No facilitator identified in this category*
Innovation deliverers / opportunity	Healthcare professionals struggle with keeping track of and implementing the multitude of guidelines due to limited capacity and time constraints	*No facilitator identified in this category*
Innovation deliverers / motivation	Healthcare professionals experience guideline fatigue	*No facilitator identified in this category*
**Implementation process domain**		
Planning	Guideline implementation plan is very concise, not concrete and/or copied from a previous guideline	*No facilitator identified in this category*
	No adequate attention/follow-up/action to guideline implementation plan/pilot implementation	
**THEME 2: Implementation knowledge and expertise**		
**Outer setting domain**		
Local attitudes	Belief that implementation occurs swiftly/automatically once a guideline is published and requires minimal resources	*No facilitator identified in this category*
**Individuals domain (roles subdomain / characteristics subdomain)**^b^		
Implementation team members / capability	Guideline committee insufficiently considers or lacks the necessary expertise to address the implementability of the guideline during its development process	*No facilitator identified in this category*
	Guideline committee lacks adequate expertise in planning/executing implementation strategies	
Innovation deliverers / capability	Healthcare professionals have insufficient implementation expertise	Train implementation (science) practitioners to improve implementation expertise
**THEME 3: Guideline characteristics: representation, evidence-base and design**		
**Innovation domain**		
Innovation source	Guideline, guideline committee and/or guideline developer predominantly reflect an (academic) medical professional perspective, giving less consideration to other interests, stakeholder perspectives and contexts (e.g. patient or general hospital perspective)	Guidelines developed by peers and endorsed by scientific organizations foster consensus and support for their use among healthcare professionals
		Acceptance of the guideline is influenced by the favorable reputation that scientific/professional organizations maintain among their associated healthcare professionals
Innovation evidence-base	Guidelines quickly become outdated due to the constant influx of new evidence and the lengthy development process	Guideline’s (perceived) credibility and evidence-base
	Insufficient conclusive evidence, resulting in weak guideline recommendations	
Innovation complexity	Multidisciplinary guidelines are harder to develop and implement due to the involvement of multiple stakeholders with different interests	Guideline recommendations that are concrete, practical, and straightforward, without unnecessary complexity
Innovation design	Guideline recommendations are formulated vaguely, as guideline committees are cautious about formulating strong recommendations, to preserve professional autonomy and avoid being held accountable by peers	Modular updating and structuring of guidelines save the guideline committee energy/time which could be re-allocated towards implementation efforts, enable faster incorporation of new insights, and make the recommendations more manageable to disseminate/implement
	Guidelines are extensive documents, difficult to understand for some end users	Guideline described from patient perspective
**Outer setting domain**		
Partnerships & connections	Not all (guideline) organizations agree with certain guideline recommendations	*No facilitator identified in this category*
**THEME 4: Partnerships and collaboration**		
**Outer setting domain**		
Partnerships & connections	Guideline organizations do not have a clear view of the similarities and collaboration opportunities regarding each other's quality initiatives	The bond of trust between specific (representatives of) guideline organizations strengthens implementation efforts
	Central and local stakeholders (e.g. insurers, healthcare professionals, healthcare facilities’ board of directors) do not collaborate effectively due to mutual distrust	Formal collaborative agreements between guideline organizations (e.g., ZE&GG program) ensure a collective commitment to implementation and provide opportunities to more easily reach multiple stakeholders
	Guideline organizations do not use all collaborative opportunities to inform stakeholders about guidelines	
**Inner setting domain**		
Relational connections	*No barrier identified in this category*	Healthcare facilities with short communication lines between healthcare professionals, support functions (IT department), and management adapt to guidelines more quickly
**THEME 5: Characteristics of guideline implementation planning, execution and evaluation strategies**		
**Innovation domain**		
Innovation design	*No barrier identified in this category*	Recurring fixed guideline publication moment
**Outer setting domain**		
Partnerships & connections	*No barrier identified in this category*	Critical sources/websites containing guideline information (e.g. Thuisarts and websites of scientific/professional organizations) are updated and aligned
**Inner setting domain**		
Structural characteristics	*No barrier identified in this category*	Aligning information technology infrastructure with the guideline
		Structural attention to guidelines in healthcare professional practice meetings (e.g. handovers, multidisciplinary consultations)
Access to knowledge & information	*No barrier identified in this category*	Implementation Agenda raises awareness within healthcare facilities about the need for implementation and their responsibility in the process
**Individuals domain (roles subdomain / characteristics subdomain)**^b^		
Implementation leads / motivation	*No barrier identified in this category*	Dedicated implementation leader
**Implementation process domain**		
Assessing needs	*No barrier identified in this category*	Through close engagement with stakeholders, guideline organizations can better understand their implementation needs and effectively facilitate them
Planning	Implementation is not addressed throughout the process, but only in the last phase	Creating a detailed step-by-step plan for the implementation process
		Addressing implementation and engaging stakeholders early on, already in the guideline development process
Tailoring strategies	Guidelines are published in different guideline databases, which makes it less user-friendly and creates uncertainty about guideline currency and quality	Ensuring easy access to guideline content for end users (through guideline database and patient information)
	Implementation strategies are not tailored to guideline end users	
Engaging	Forgotten/late engagement of stakeholders in the implementation planning/execution	Empowering patients through the development of guideline information specifically for patients
		A communication advisor is involved and assists implementation
Doing	No standardized guideline development and implementation process	*No facilitator identified in this category*
Reflecting & evaluating	Insufficient good quality data on the success of implementation	Audit & feedback benchmark information is useful to support implementation and evaluation
	Balancing the need for data collection for measuring implementation success against the perceived burden of administration is challenging	
**THEME 6: Characteristics of healthcare professionals: need, capability, opportunity and motivation**		
**Inner settings domain**		
Communications	Open communication across hierarchical structures (e.g. work experience, nurse-physician relationship) about changing practice is perceived as challenging	*No facilitator identified in this category*
Culture	*No barrier identified in this category*	Healthcare facility and healthcare professionals are learning-centered
		Culture of adhering to guidelines ingrained within the profession
**Individuals domain (roles subdomain / characteristics subdomain)**^b^		
Innovation deliverers / need	*No barrier identified in this category*	There is a strong demand among healthcare professionals for a specific guideline (e.g. there is a high level of uncertainty)
Innovation deliverers / capability	Certain medical professional groups are not yet accustomed to using guidelines	*No facilitator identified in this category*
	Healthcare professionals are hesitant to (de-)implement out of fear of causing harm to patients	
	Healthcare professional’s lack of confidence/knowledge/skills to execute guideline	
	Healthcare professionals’ fear of going against patients’ expectations/wishes	
Innovation deliverers / motivation	Healthcare professionals cling to old habits and routines	Healthcare professionals are intrinsically motivated to improve and deliver good care
	Healthcare professionals experience cognitive dissonance between their past/current practices and the guideline-recommended practices	
	Healthcare professionals perceive guidelines as restricting their professional autonomy	
**THEME 7: Legal and regulatory compliance**		
**Outer setting domain**		
Policies & laws	Regulations (e.g. volume standards, rules about fair cooperation between healthcare facilities from the Authority for Consumers and Markets (ACM)) hinder implementation	Healthcare professional must justify themselves to a disciplinary or incident review board due to an incident/complaint and undergo assessment to determine if they have worked in accordance with the professional standard
	Conflicts between different guidelines/protocols on the same topic	
External pressure	Guideline organizations have insufficient mandate/power to push implementation, compared to significant healthcare professional autonomy	Health and Youth Care Inspectorate (IGJ) can use its authority/power to push implementation in healthcare facilities

Implementation determinants are classified according to the updated CFIR [29]. Definitions and detailed descriptions of the updated CFIR concepts are presented in the additional files of Damschroder et al. (2022)

^a^Implementation determinants that did not fit in the original updated CFIR were classified and added in *italicized* text

^b^In the individuals domain , we coded the relevant characteristics for each identified role, following the recommendation of Damschroder et al. (2022)

1. Healthcare demand and resource availability

Representatives unanimously recognized that barriers and facilitators related to healthcare demand and resource availability influenced guideline implementation. At the health system level, increasing demand for care, driven by factors like an aging population, combined with care resource constraints, such as staff shortages, forced compromises in care quality, accessibility, and affordability. As a result, the high standards outlined in guidelines were not always attainable. However, representatives noted that this pressure also heightened the focus on prioritizing appropriate care (described in guidelines), de-implementation of low-value care and effective implementation:

‘*We face challenges in healthcare on all fronts, including accessibility, staffing, and affordability. When it comes to making critical decisions about where to allocate our limited workforce, we prefer to focus on care that we know adds value for the patient.*’ – Representative 1, national organization

Another mentioned macro-level barrier was the weak link between care quality and reimbursement, as the system is still dominated by fee-for-service payment structures instead of quality-based payment models. Some representatives noted concerns among healthcare facilities and professionals about revenue reduction when (de-)implementing or reallocating care across professions, as recommended in guidelines. Representatives also encountered issues with temporary funding of guideline implementation projects, leading to unsustainable implementation or continued use of outdated guidelines or tools that had not been properly de-implemented.

At the level of guideline organizations and guideline committees, representatives observed a predominant allocation of resources (time, staff, funding) to developing and revising guidelines, rather than to their dissemination and implementation. Guideline committees often lacked time for implementation planning, resulting in concise, non-specific and copied implementation plans and insufficient follow-up for their practical execution.

‘*[The implementation plan] is actually made entirely at the end of the guideline process and there is a certain budget allocated for creating it. We almost always run out of time, leaving very little time to truly focus on implementation. It becomes more of a checkbox exercise and is often filled in without much thought. And subsequently, scientific organizations, I believe, also don't look into it enough.*’ – Representative 10, guideline developer

Representatives noted that guideline funders varied in prioritizing implementation as an integral part within guideline development. Guideline developers often had to request separate funds for developing implementation strategies, which were not always approved. Some guideline developers, often with a large number of members (healthcare professionals), allocated dedicated resources and teams for planning and executing implementation strategies. However, most guideline organizations reported having limited or no capacity for implementation due to the sheer volume of guidelines, limited resources and prioritization of more urgent and mandatory tasks (e.g., quality programs, registrations):

‘*That it's impossible to continuously work on implementing 3,000 guidelines, goals, and who knows what else, while constantly knowing the progress and what is or isn’t effective. So it’s all about choices, choices, choices.*’ – Representative 35, governmental agency

Consequently, the responsibility for planning and executing implementation strategies typically fell on the guideline committee members, often relying on their motivation and voluntary efforts.

Representatives mentioned that healthcare facilities and professionals also struggled to prioritize and implement guidelines due to the multitude of quality initiatives and guidelines available, limited capacity and funding, and the compatibility of requested organizational changes. According to a few representatives, this caused guideline fatigue among healthcare professionals. Here, financial compensation was seen as an important facilitator, allowing healthcare facilities and professionals to dedicate time for implementation tasks.

2. Implementation knowledge and expertise

Various representatives noted the widespread misconception that implementation occurs swiftly and automatically once a guideline is published, and that implementation requires minimal resources. Furthermore, representatives observed that some guideline committees lacked the consideration and expertise to address guideline implementability and actual implementation. Often, there was a lack of understanding of barriers hindering compliance with specific guidelines across different settings (from patient to health system).

‘*The success of implementation also depends on how well the guideline is constructed. Has implementability been adequately considered in that guideline? Is there sufficient capacity to make changes? Is the physician population ready to tackle things differently? Is there enough funding? Is the hospital willing to invest? So, those are all aspects that you actually need to discuss very thoroughly when [developing] a guideline. And I see that that is not always the case.*’ – Representative 27, national organization

To address these barriers related to implementation knowledge and expertise, some guideline developers formed dedicated implementation teams or added implementation experts, like communication specialists, to their guideline committees.

Representatives also noted a frequent lack of implementation expertise among healthcare professionals and facilities. ‘*They don't really get that from their training, so they sometimes find it very difficult to do*’ (Representative 12, national organization). Those with expertise were frequently overburdened. To address this, several guideline organizations invested in training implementation (science) practitioners.

3. Guideline characteristics: representation, evidence base and design

Representatives mentioned that representation of stakeholders within guidelines affected implementation. Some observed that guidelines, guideline committees and/or involved guideline developers often reflected an (academic) medical professional perspective. They expressed concerns that the content and design of certain guidelines insufficiently considered broader stakeholder interests and contexts. Specifically, some guidelines overlooked the patient perspective and misaligned with the diversity in patient populations or care capabilities, for example in non-academic hospitals or clinics. Furthermore, certain guidelines focused too narrowly on medical and condition-specific possibilities, missing broader considerations like costs and care organization implications. Representatives said that these aspects led to some guideline organizations not fully endorsing the guidelines, slow or incomplete implementation in healthcare facilities, and mixed support from guideline developers’ members:

‘*But we sometimes still see a gap between the scientific organization and actual practice. Scientific organizations often consist of enthusiastic people who want to collaborate and prioritize quality highly. So, even if such a scientific organization is at the table, it doesn't mean that the entire professional group stands behind it*.’ – Representative 23, governmental organization

Another challenge that representatives encountered was reaching consensus on multidisciplinary guidelines, as the involvement of many stakeholders with differing interests complicated and slowed the development and implementation process. On the other hand, representatives noted that guidelines developed by medical peers and endorsed by reputable guideline developers were more likely to gain acceptance and support among healthcare professionals. Another facilitator mentioned was writing guidelines from the patient's perspective (patient journey), which fostered a sense of joint effort for patients among end users.

The evidence supporting guideline recommendations was considered another key determinant. Representatives noted that guidelines quickly became outdated due to the constant influx of new evidence and lengthy development processes, hindering their effective use. The weak formulation of guideline recommendations, due to insufficient conclusive evidence, also negatively impacted adoption. A representative noted that guideline recommendations were sometimes deliberately formulated vaguely, as guideline committees wanted to preserve professional autonomy. Conversely, representatives mentioned that concretely formulated recommendations and strong supporting evidence helped convince end users to adapt their practice:

‘*The guidelines that implement most easily are simply the ones where the evidence is very clear. … Then you don't even need to create a guideline, so to speak. If the outcome is clear, then there is no problem at all in implementing the guideline. None at all*.’ – Representative 31, guideline developer

The third influential guideline characteristic was its design. Its recently introduced modular updating and structuring enabled faster incorporation of new insights, potentially saving the guideline committee time and energy that could be redirected towards implementation. However, representatives noted that guidelines remained extensive, often unread, and difficult to navigate for some users (e.g., patients, care assistants).

4. Partnerships and collaboration

An overarching health system challenge affecting implementation was mutual distrust among central and local stakeholders (e.g., healthcare facilities, governmental agencies, health insurers), driven by negative perceptions of each other’s interests. Representatives observed that this distrust hindered effective collaboration on guideline implementation:

‘*Yeah, and if I trust you, then I want to help you get that done. … Collaborating isn't saying, 'you have to do that'. Collaborating is thinking together, ‘what do we have, what needs to happen, and what can I do?’ And we're really not there yet, are we? … That lack of collaboration, lack of trust, it's in all parties.*’ – Representative 32, guideline developer

Representatives saw formal collaborative agreements, such as the Outline Agreement on Medical Specialist Care (HLA-MSZ) and the Health Care Evaluation and Appropriate Use (ZE&GG) program – an initiative where patients, healthcare professionals, facilities, insurers, and the government work together to deliver the proven best care for patients – as facilitators. Representatives argued that these agreements not only facilitated easier access to other stakeholders, but also improved collaboration, built trust, and ensured collective commitment to implementation: ‘*And that's what the ZE&GG program is currently striving to achieve: to emphasize that everyone has a role to play in this endeavor*’ (Representative 14, governmental agency).

However, some representatives observed missed opportunities for collaboration between organizations. They mentioned that a lack of awareness about shared goals and collaboration opportunities between their quality initiatives and restrictive collaborative regulations limited joint efforts.

At the level of healthcare facilities and professionals, some representatives noted that shorter communication lines between healthcare professionals, support functions (IT department) and management – often found in smaller facilities such as clinics – facilitated quicker adaptation to guidelines.

5. Characteristics of guideline implementation planning, execution and evaluation strategies

Regarding implementation planning, representatives noted significant variations in guideline development and implementation policies across guideline organizations. This included differences in guideline names, characteristics, and development and implementation processes. This lack of standardization was viewed by some as a barrier:

‘*Then you should really take a moment to consider: how can we ensure that we streamline it as much as possible? So that it's easier for everyone to understand. All those different definitions of guideline types, the entire process, all those different entities involved. It should be more standardized.*’ – Representative 5, national organization

Representatives also observed that implementation was often considered only in the final phase of guideline development instead of from the outset and throughout. Additionally, stakeholders, including end users, were sometimes engaged late or overlooked in implementation planning and execution, resulting in missed opportunities for tailored implementation. Another identified barrier was that implementation strategies (tools) were not always tailored to meet end users’ needs: ‘*it [implementation tool] was introduced, and then no doctor looked at it. Simply because it didn’t fit into their work and they didn’t have time for it*’ (representative 1, national organization). Various representatives noted that guideline databases were particularly untailored. While commonly used and valued for dissemination, storing different types of guidelines in separate databases reduced user-friendliness and introduced uncertainty about guideline quality and currency. Some also learned from healthcare professionals that database search options and other features did not meet their information needs.

Representatives identified facilitators that complement the previously stated barriers. They observed that addressing implementation and engaging stakeholders early in the development process could enhance implementation effectiveness. Close stakeholder engagement provided guideline organizations with insights into implementation feasibility, barriers and end users' needs. Additionally, creating a detailed step-by-step plan could streamline both development and implementation processes: ‘*so, we've created several good process descriptions. I find that beneficial*’ (representative 15, guideline developer).

Regarding implementation execution, representatives highlighted several used strategies that, in their experience, specifically enhanced implementation. They emphasized the importance of having a dedicated implementation leader, as well as a communication specialist to aid in developing effective implementation strategies. A representative noted that establishing recurring fixed guideline publication moments (e.g., annually at the professional conference) set clear expectations for end users and improved dissemination. Ensuring guideline accessibility was also emphasized, including publishing in a guideline database and updating and aligning information sources like patient and professional websites. At the healthcare facility level, representatives highlighted aligning information technology, such as electronic patient records, with guidelines as an effective strategy. Another perceived effective strategy was ZE&GG’s implementation agenda, which raised awareness in healthcare facilities about the recommendations that needed to be implemented. At the patient level, developing tailored guideline information to empower patients was emphasized as an important strategy.

With regard to implementation evaluation, representatives indicated that collecting and presenting audit and feedback information was valuable for identifying areas for improvement and driving care enhancements. However, some noted a lack of high-quality data on implementation performance, leaving the state of practices and undesirable practice variations unclear. Additionally, healthcare facilities and professionals sometimes dismissed the data as inaccurate. Finding a balance between the benefits (identifying areas of improvement) and burdens (administrative workload) of data collection for evaluation was considered challenging and required compromises.

6. Characteristics of healthcare professionals: need, capability, opportunity and motivation

Representatives identified implementation determinants related to characteristics of healthcare professionals. With regard to need, representatives noted that a strong, bottom-up demand for a specific guideline boosted its implementation. High uncertainty, risk of harm and urgency surrounding conditions, such as with COVID-19, created a pressing need for clear, reliable recommendations among healthcare professionals.

In terms of capability, representatives emphasized that healthcare professionals' (interpersonal) skills and (implementation) knowledge significantly impacted implementation. According to representatives, a considerable number of professionals lacked the necessary knowledge, confidence and expertise for effective guideline implementation. New guidelines often required more specialized skills, which could be daunting. Another factor influencing implementation was that some medical professional groups, such as nurses, were relatively new to using guidelines, unlike more experienced groups like GPs with a long history of guideline use. Additionally, representatives noted that the introduction of new or revised guidelines, particularly those aimed at discontinuing low-value care, often met with hesitation from professionals due to concerns about potential patient harm, conflicting expectations, or resistance during consultations: ‘*but when a patient makes a request to you, it's very difficult to say no*’ (Representative 25, health insurer).

Regarding implementation opportunities for healthcare professionals, representatives suggested that a learning-centered approach in healthcare facilities could aid implementation, by allowing healthcare professionals to learn without immediate consequences. However, strong hierarchical structures based on experience, seniority, and nurse-physician dynamics could impede the open communication needed for learning and implementing changes.

Regarding motivation, many representatives emphasized that healthcare professionals were intrinsically motivated to improve care, which should be encouraged and harnessed by guideline organizations. ‘*I would say, within nursing, that a promoting factor is their true passion for the profession and strong motivation to deliver quality care*’ (Representative 4, guideline developer). However, representatives frequently experienced that healthcare professionals clung to old habits, making change difficult after years of established routines. Cognitive dissonance — the mental discomfort healthcare professionals experience upon realizing that their past or current practices conflict with guideline-recommended practices — was another identified barrier. Representatives also mentioned that some healthcare professionals perceived guidelines as hindering personalized care and limiting their professional autonomy.

7. Legal and regulatory compliance

Representatives identified various barriers and facilitators related to legal and regulatory compliance. They mentioned that conflicting guidelines and regulations, such as European guidelines, volume standards or regulations from the Authority for Consumers and Markets (ACM) sometimes hindered implementation:

‘*We’re going to see how we measure up against the European professional association, which is holding its conference here in Amsterdam in September and will be presenting all their new guidelines.*’ – Representative 33, guideline developer

Furthermore, several representatives from governmental agencies and health insurers felt that their organizations lacked the mandate to drive implementation compared to healthcare professionals. They respected professional autonomy but desired additional means to enforce recommendations when necessary. The Health and Youth Care Inspectorate (IGJ), the governmental agency that supervises the quality and safety of healthcare and youth care services in the Netherlands, was recognized as having this authority to enforce implementation. However, the IGJ focused on broader quality and safety aspects beyond guidelines (e.g., organizational culture and working environment) and pursued its own improvement objectives. An identified facilitator was the legal obligation for healthcare professionals to follow guidelines. In case of serious incidents or complaints, healthcare professionals had to justify their actions to a review board and undergo assessment, which served as an extrinsic motivator to align their practices with guidelines:

‘*A negative situation is when you experience a complication and you have to appear before the disciplinary board or incident review board because you need to justify yourself. Yes, those who have experienced it once will definitely read the guidelines the next time. But that's only one in so many cases.*’ – Representative 17, guideline developer

## Discussion

The implementation of guidelines is a critical process that directly impacts the quality of healthcare delivery. However, the successful translation of these guidelines into practice faces numerous barriers while also benefiting from various facilitators. This qualitative study offers a comprehensive overview of the barriers and facilitators encountered by representatives of Dutch guideline organizations in their efforts to support the implementation of guidelines. We identified 45 barriers and 35 facilitators, categorized into seven overarching themes. Representatives encounter barriers and facilitators predominantly on the level of implementation team members (guideline committees/guideline organizations), those delivering care (healthcare professionals), and the outer setting in which guideline organizations operate.

Important health system-wide barriers to effective guideline implementation include the constrained availability of resources for both care delivery and implementation, compounded by the growing demand for care and the multitude of quality recommendations, initiatives and tasks. These determinants make it challenging for guideline organizations and end users to prioritize guideline implementation. Such barriers have also been identified in previous research, including two meta-reviews on global implementation barriers and facilitators [31, 33], as well as more targeted empirical studies examining implementation experiences of international guideline organizations, Dutch hospitals and Dutch general practitioners [16, 30, 44]. As workforce shortages, care demand and the number of care quality recommendations continue to grow [9, 45, 46], achieving the optimal care standards outlined in guidelines is likely to become even more difficult in the years ahead. This urges the need to rethink guideline policies and nationwide care quality strategies. With advancements in artificial intelligence potentially enabling efficient real-time guideline development [47], we propose to strategically invest in this innovative approach, coupled with a gradual reallocation of efforts towards guideline contextualization and implementation. Blume et al. (2017) [27], for instance, recommend that guideline developers should grade the relative relevance of guideline recommendations to better align with real-world constraints, so that end users can balance the delivery of guideline-recommended care against the available resources. Furthermore, we suggest structurally embedding implementation as a core objective of the guideline development process, supported by dedicated resources. Lastly, stakeholders could reassess the prioritization of guideline implementation in the multitude of their care quality tasks. These strategies may tackle the aforementioned barriers, improving the applicability, implementation and impact of guidelines on healthcare outcomes.

The identified determinants in this study also highlight other systemic issues. The determinants of trust, collaboration, stakeholders’ enforcement power and the weak link between care quality and reimbursement, fit within broader current criticisms of the ‘managed competition’ model underlying the Dutch healthcare system. This model, in which healthcare facilities negotiate with health insurers on price, volume and quality of care, aims to improve efficiency, reduce central governance and enhance accessibility of good quality health services at acceptable societal costs [48]. Critics, however, argue that market mechanisms have been overextended. Competition and cost-savings have become more important than collaboration and healthcare quality. Furthermore, a power conflict exists between health insurers and healthcare facilities and trust between stakeholders is low [49–51]. Boosted by the increased collaboration during the COVID-19 pandemic and recent collaborative initiatives like the Integral Care Agreement [52] and ZE&GG program, there seems to be growing support for enhanced stakeholder cooperation to stimulate appropriate care, moving beyond a strict managed competition model. Our results show that stakeholders in guideline implementation are interdependent, as each can address only part of the identified determinants. Greater collaboration between guideline organizations and end users may therefore offer key opportunities for improvement. We recommend to collectively focus implementation resources and actions on specific, robustly evidence-based recommendations with significant positive impact. This national implementation agenda setting, an extension of ZE&GG’s Implementation Agenda [53], could foster alignment of implementation efforts, trust between stakeholders, and delivery of appropriate care as outlined in the guidelines.

Our study also highlights conflicting regulations, legal obligations and the balance between professional autonomy and guideline organizations’ enforcement mandates as important determinants. These determinants stress the intricate interplay between intrinsic and extrinsic motivators in enhancing change. While extrinsic motivators are commonly employed to target ‘laggards’ [54], some guideline organizations in our study proposed expanding their use due to the current pressures on healthcare and slow implementation progress. Extrinsic motivators may enhance guideline adherence and prompt quick changes, but they may also cause resistance, lead to superficial compliance, undermine professional autonomy, fail to increase change capacity, divert resources from other urgent issues and require significant resources for implementation and monitoring [55–57]. Sustainable improvement may therefore require strategies that foster intrinsic motivation and integrate guideline implementation into the culture and daily practice of healthcare professionals and facilities, alongside extrinsic motivators that align with these end users’ intrinsic values [56, 57].

Our results indicate that because of growing pressures on the healthcare system, stakeholders are increasingly interested in what is written in guidelines regarding what low(er)-value care to remove, replace, reduce or restrict. Previous research highlights differences between the act of implementing versus de-implementing, including unique determinants and effective strategies [58–60]. Although our study does not specifically explore these differences, we identify several barriers to de-implementation that highlight its complexity and distinct challenges. These include established routines, cognitive dissonance, and concerns about potential patient harm, conflicting expectations and potential revenue loss. Guideline organizations should consider the differing determinants when selecting and tailoring strategies for implementation versus de-implementation, as certain strategies – such as holding healthcare professionals accountable when prescribing low value care [61] – may be especially effective for de-implementation. Additionally, when revising guidelines, outdated recommendations and associated implementation tools must be explicitly de-implemented to avoid continued use.

Another important finding is the gap in implementation knowledge and expertise among guideline committees and end users. The expectations of effective guideline implementation with minimal activities and resources contrast sharply with the complex, unpredictable, resource-intensive realities of actual implementation. This misconception has been noted in previous studies among national and local guideline implementation initiatives as well as broader governmental policy implementation [3, 62–64]. Literature stresses the need to integrate implementation science and expertise into implementation teams and initiatives [18, 64, 65]. Alongside current efforts to improve implementation knowledge and expertise, such as appointing communication specialists and training implementation science practitioners, stakeholders would benefit from guidance from a central implementation team. This team could support context-specific implementation by leveraging implementation knowledge, insights from best practices and adaptable implementation tools. This approach would prevent stakeholders from reinventing the wheel and potentially lead to more effective implementation efforts.

Lastly, representation, evidence base and design are considered key guideline characteristics in successful guideline development and implementation. Our results confirm that guidelines overly focused on certain stakeholders can alienate other perspectives and interests, which could affect guideline acceptance, relevance and applicability across various settings [66, 67]. However, we also found that involving numerous stakeholders can delay the guideline development and implementation process. This corroborates previous Dutch research on guideline development, emphasizing the importance of balancing inclusiveness with efficiency [68]. The study also supports previous research [69, 70] showing that outdated or weakly evidence-based recommendations hinder adoption, underscoring the importance of regular guideline updates to align with the latest scientific findings. Modular updates are viewed as efficient for maintaining relevance and potentially freeing up time for implementation. However, it remains uncertain whether the time saved is actually redirected towards implementation or instead used for developing and revising other guideline modules, which further challenges end users in staying up-to-date.

### Strengths and limitations

A key strength of this study is the great diversity of participating guideline organizations. This diversity provided a broad representation of various roles, perspectives, professions, and specializations, enriching the research with multiple viewpoints. However, it is important to note that the participating organizations may have had a greater interest in implementation, potentially leading to selection bias. While thematic saturation was achieved, the interviewed organizations may not fully represent the entire spectrum of guideline organizations and their implementation practices.

In asking the representatives about the barriers and facilitators, we adopted an open and exploratory approach. We did not review the entire list of potential barriers and facilitators provided by CFIR with each representative, nor did we cross-check all the barriers and facilitators mentioned by the representatives with others. Consequently, certain determinants may have been missed or may have been considered more or less important. Given that determinants vary across different settings, guidelines and even individual recommendations, tailored and barrier-driven implementation strategies focused on key recommendations are necessary to enhance adherence of specific guidelines in practice. Nevertheless, we are confident that our interview method has enabled us to capture the most significant guideline-transcending barriers and facilitators.

A final limitation is that the representatives also mentioned determinants they believed were significant from the perspective of other stakeholders, such as healthcare professionals. In this study, it was difficult to verify the accuracy of these interpretations. However, some representatives worked closely with other stakeholders or held multiple roles within different organizations. For example, some representatives were also working as healthcare professionals or had previously worked in that capacity. Further research is needed to explore and verify the perceived guideline implementation barriers and facilitators among other stakeholders.

## Conclusion

Guideline organizations play an important role in facilitating the implementation of guidelines in practice. This study offers valuable insights into the complex dynamics of barriers and facilitators perceived by representatives of guideline organizations in nationwide guideline implementation. The findings help explain why healthcare professionals and healthcare facilities may (not), slowly or inconsistently adhere to guideline recommendations in practice. Findings from this study also provide considerations and guidance for policymakers to re-evaluate and improve nationwide quality and guideline implementation policies, to eventually improve clinical practice and health outcomes for patients.

## Supplementary Information

Supplementary Material 1: Completed COREQ Checklist.

Supplementary Material 2: Interview guide.

Supplementary Material 3: Coding tree.

Supplementary Material 4: Implementation barriers and facilitators identified by representatives of guideline organizations, classified according to the updated CFIR framework.

## Data Availability

The interview data that were used and analyzed during the current study are available from the corresponding author upon reasonable request.

## Abbreviations

CFIR: Consolidated Framework for Implementation Research
COREQ: Consolidated criteria for reporting qualitative research
ZE&GG program: Zorgevaluatie & Gepast Gebruik programma (Health Care Evaluation and Appropriate Use program)
