# Supplementary material for: Nationwide guideline implementation: a qualitative study of barriers and facilitators from the perspective of guideline organizations
Source: BMC Health Serv Res. 2025 Jan 27;25:150. doi: 10.1186/s12913-025-12270-2 (PMC11771117; doi:10.1186/s12913-025-12270-2)
Supplement: Supplementary file 1 — Supplementary Material 1: Completed COREQ Checklist. [file 12913_2025_12270_MOESM1_ESM.docx]

Supplementary material 1. Completed COREQ (COnsolidated criteria for REporting Qualitative research) Checklist

| **Topic** | **Guide Questions/description** | **Details (manuscript page number, if reported)** |
| --- | --- | --- |
| Domain 1: Research team and reflexivity | | |
| Personal characteristics | | |
| 1. Interviewer/ facilitator | Which author(s) conducted the interview or focus group? | Interviews were conducted by one or two researchers:  AT, TB, AG. (p. 8) |
| 2. Credentials | What were the researcher’s credentials? (e.g. PhD, MD) | AT: MSc.  AG: BSc, received MSc after completing the internship that this study was part of.  TB: BSc, received MSc after completing the internship that this study was part of.  IvB: PhD  DD: Prof.Dr.  MdB: Prof.Dr.  HM: PhD |
| 3. Occupation | What was their occupation at the time of the study? | AT: PhD researcher  HM: assistant professor  TB: MSc student/intern  AG: MSc student/intern  IvB: advisor and researcher  DD: professor and chief scientific officer  MdB: professor and director |
| 4. Gender | Was the researcher male or female? | Female: AT, HM, AG, IvB, DD, MdB  Male: TB |
| 5. Experience and training | What experience or training did the researcher have? | The interviewers (AT, TB, AG) had a background in health policy studies, were trained in interviewing techniques and had interview experience. (p. 8) |
| Relationship with participants | | |
| 6. Relationship established | Was a relationship established prior to study commencement? | No prior relationship was established between the researchers and participants |
| 7. Participant knowledge of the interviewer | What did the participants know about the researcher? (e.g. personal goals, reasons for doing the research) | Participants knew the occupations of the interviewers, where they worked and the purpose of the research |
| 8. Interviewer characteristics | What characteristics were reported about the interviewer/facilitator? (e.g. bias, assumptions, reasons and interests in the research topic) | DD and IvB, besides their roles as researchers, are employed by Zorginstituut Nederland, a guideline organization. While this may introduce potential bias or conflicts of interest, we have mitigated this by extensively addressing and discussing the matter in our group discussions on study design and result interpretation. (p. 30) |
| Domain 2: study design | | |
| Theoretical framework | | |
| 9. Methodological orientation and theory | What methodological orientation was stated to underpin the study? (e.g. grounded theory, discourse analysis, ethnography, phenomenology, content analysis) | We employed framework analysis, using the updated Consolidated Framework for Implementation Research (CFIR), and thematic analysis to guide our data analysis and synthesis. (p. 9) |
| Participant selection | | |
| 10. Sampling | How were participants selected? (e.g. purposive, convenience, consecutive, snowball) | The study population consisted of representatives of Dutch guideline organizations. We used purposive sampling methods to recruit a broad sample of these representatives. Eligible guideline organizations were scientific/professional organizations, knowledge institutes, governmental agencies, health insurers, patient organizations and other national (umbrella) organizations that developed guidelines, published them and/or actively supported their use in clinical practice. Representatives were recruited based on their understanding of their organization's role or their own direct involvement in guideline implementation. We recruited potential participants through contact information obtained from guideline organization websites, contacts of the research team, as well as snowball sampling, where interviewed representatives recommended others. (p. 7-8) |
| 11. Method of approach | How were participants approached? (e.g. face to face, telephone, mail, e-mail) | Participants were contacted via email or telephone. (p. 8) |
| 12. Sample size | How many participants were in the study? | A total of 35 participants from 24 different guideline organizations were interviewed. (p. 11) |
| 13. Non-participation | How many people refused to participate or dropped out? Reasons? | Numbers of refusals were not recorded. |
| Setting | | |
| 14. Setting of data collection | Where was the data collected? (e.g. home, clinic, workplace) | Interviews were held via videoconference or in-person (e.g. at their workplace) based on participant preference. (p. 8) |
| 15. Presence of non-participants | Was anyone else present besides the participants and researchers? | Only the researchers and participants were present. |
| 16. Description of sample | What are the important characteristics of the sample? (e.g. demographic data, date) | Guideline organization, occupation(s) and role in relation to guideline implementation. (p. 11) |
| Data collection | | |
| 17. Interview guide | Were questions, prompts, guides provided by the authors? Was it pilot tested? | Interview topic guide with prompts (Appendix 2) was developed and used during the interviews. The topic guide was not pilot tested, but it was extensively reviewed within the research team. (p. 8 and Appendix 2) |
| 18. Repeat interviews | Were repeat interviews carried out? If yes, how many? | No repeat interviews were required. |
| 19. Audio/visual recording | Did the research use audio or visual recording to collect the data? | Interviews were audio recorded. (p. 8) |
| 20. Field notes | Were field notes made during and/or after the interview or focus group? | Field notes were made during the interview. |
| 21. Duration | What was the duration of the interviews or focus group? | Interviews lasted between 30-100 minutes. (p. 8) |
| 22. Data saturation | Was data saturation discussed? | Data collection continued until no new themes emerged, signifying data saturation. (p. 8) |
| 23. Transcripts returned | Were transcripts returned to participants for comment and/or correction? | Participants received an interview summary for commenting (member checking). (p. 8) |
| Domain 3: analysis and findings | | |
| Data analysis | | |
| 24. Number of data coders | How many data coders coded the data? | Initially, two researchers (AT and either TB or AG) independently coded the first eight interviews to align coding. Subsequently, one researcher (TB, AG, or AT) coded the rest, cross-checked by a second researcher (AT or HM). (p. 9) |
| 25. Description of the coding tree | Did authors provide a description of the coding tree? | The final coding tree is provided in Appendix 3. (p. 9 and Appendix 3) |
| 26. Derivation of themes | Were themes identified in advance or derived from the data? | Interview transcripts were analyzed using the principles of framework analysis. The updated Consolidated Framework for Implementation Research (CFIR) guided our analysis. We applied deductive coding to the data. We developed an initial codebook based on the implementation domains and determinants from the updated CFIR to categorize the barriers and facilitators that representatives identified. Furthermore, open coding was used to capture interesting aspects that emerged from the data, such as additional barriers and facilitators. The codebook was updated iteratively throughout the process.  After systematically categorizing the identified barriers and facilitators using the updated CFIR and organizing them in a data extraction template (Excel), we conducted a further analysis to explore their interactions and dynamics. This involved examining patterns, connections and influences between barriers and facilitators, as well as across different stakeholders and CFIR domains. Through this additional thematic analysis, we identified seven themes of barriers and facilitators, extending beyond the original CFIR domains. To provide a clearer understanding of the interactions and dynamics between determinants, the results are presented according to these themes. (p. 9-10) |
| 27. Software | What software, if applicable, was used to manage the data? | Coding was conducted using MAXQDA (version 2022). (p. 9) |
| 28. Participant checking | Did participants provide feedback on the findings? | Participants did not provide feedback on the findings. All representatives received a summary of their interview for member checking afterward. (p. 8) |
| Reporting | | |
| 29. Quotations presented | Were participant quotations presented to illustrate the themes/findings? Was each quotation identified? (e.g. participant number) | Participant quotes were presented to illustrate te findings throughout the results section. Furthermore, Appendix 4 provides a comprehensive list of the barriers and facilitators, supported by illustrative quotes. Each quote is identified through a participant number and his/her corresponding type of guideline organization. (p. 12-22 and Appendix 4) |
| 30. Data and findings consistent | Was there consistency between the data presented and the findings? | We strived to present the study findings with clarity and consistency, aiming to accurately reflect the collected data. |
| 31. Clarity of major themes | Were major themes clearly presented in the findings? | Yes, major themes are clearly presented throughout the results section, both in text (headings) and in a table. (p. 11-22, Table 2) |
| 32. Clarity of minor themes | Is there a description of diverse cases or discussion of minor themes? | Yes, diverse cases and minor themes are clearly presented throughout the results section, both in text and in a table. (p. 11-22, Table 2) |
