# Supplementary material for: Nationwide guideline implementation: a qualitative study of barriers and facilitators from the perspective of guideline organizations
Source: BMC Health Serv Res. 2025 Jan 27;25:150. doi: 10.1186/s12913-025-12270-2 (PMC11771117; doi:10.1186/s12913-025-12270-2)
Supplement: Supplementary file 3 — Supplementary Material 3: Coding tree. [file 12913_2025_12270_MOESM3_ESM.docx]

**Supplementary material 3. Final coding tree**

**Code System**

| Current state of implementing guidelines |
| --- |
| Value of guidelines as bridge between science, policy & practice |
| Solutions/ideas for implementation problem |
| Barriers |
| Implementation process domain |
| Planning - implementation plan concise/not concrete/copied |
| Bad quality of pilot implementation |
| Planning - implementation plan not realized |
| Results pilot implementation not used for actual implementation |
| Planning – implementation is last step, not throughout development process |
| Tailoring strategies - implementation strategies don't match end users |
| Innovation/strategy doesn’t fit with workflow |
| Tailoring strategies - guideline database not practical/user friendly |
| Developers submit guidelines to different databases |
| Register is too bureaucratic |
| Engaging - forgotten/too late engagement stakeholders |
| Doing - no standardized development + implementation process |
| Reflecting & evaluating - lack of good/transparent implementation data |
| Reflecting & evaluating – audit & feedback quality vs registration burden |
| Individuals domain |
| Implementation team x opportunity - members don’t have capacity/time to plan/execute strategies |
| Experience/perception that implementation costs too much time |
| Implementation team x capability - Guideline committee does not consider implementability |
| Insufficient attention to consequences implementation for organization of care |
| Implementation team x capability - insufficient expertise in planning/execution implementation |
| Implementation team x motivation - committee members are not motivated to implement |
| Innovation deliverers x capability - professions are not used to using guidelines |
| Innovation deliverers x capability - fear of patient harm/defensive medicine |
| Innovation deliverers x capability - professionals' lack of implementation knowledge/expertise |
| Innovation deliverers x capability – healthcare professional's lack of confidence/skill to execute guideline |
| Innovation deliverers x capability - fear of going against patients' expectations/wishes |
| Innovation deliverers x opportunity - too many guidelines to keep track of/implement |
| Insufficient health professionals' time |
| Innovation deliverers x motivation - guideline fatigue |
| Innovation deliverers x motivation - old habits and routines |
| Innovation deliverers x motivation - cognitive dissonance healthcare professionals |
| Innovation deliverers x motivation - notion that guidelines limit practice freedom |
| Inner setting domain |
| Communications - open feedback across hierarchies perceived as difficult |
| Relative priority - end users need to prioritize between quality initiatives |
| Too many programs for healthcare facilities to comply with |
| Too many guidelines to implement |
| Healthcare professionals have insufficient implementation and execution capacity and funding |
| Outer setting domain |
| Local attitudes - notion implementation happens fast/automatically |
| Local conditions - increasing care demand & limited resources |
| Partnerships & connections - no collaboration between guideline organizations about similar guidelines |
| Partnerships & connections - lack of trust/collaboration between stakeholders |
| Partnerships & connections – guideline organizations do not use all opportunities to inform stakeholders  about guidelines |
| Partnerships & connections - not all guideline organizations are in agreement about guideline |
| Policies & laws - conflict guidelines and volume standards |
| Policies & laws - conflict between different guidelines |
| Financing |
| Fear of reduced revenue |
| No standard budget for implementation |
| Financiers impose different requirements about implementation |
| No sustainable resources/projects for number of years |
| Maintenance plan implementation products not parallel to guideline |
| No resources/difficult to de-implement obsolete guideline |
| Care quality and reimbursement are currently not/hardly linked |
| What to do with mildly substantiated guidelines |
| Need to be able to deviate when substantiated |
| External pressure - guideline organization doesn't have implementation instrument of power |
| External pressure - relative priority development vs implementation guideline |
| External pressure - great professional autonomy healthcare professionals |
| Capacity/resources (+) |
| Stakeholders (are) not (able to) make use of commentary round |
| Innovation domain |
| Innovation source - guideline predominantly reflects medical/professional/academic perspective |
| Innovation source - guideline committee does not reflect actual user |
| Implementation team – guideline developer doesn't reflect members |
| Innovation evidence-base - guidelines are outdated |
| Innovation evidence-base - lack of evidence so weak guideline |
| Innovation complexity - multidisciplinary guideline harder to implement |
| Innovation design - caution to formulate strong recommendations |
| Innovation design - GRADE makes process and guidelines complex |
| Innovation design - guideline extensive, complex document |
| Facilitators |
| Implementation process domain |
| Assessing needs – through close engagement with stakeholders, better understanding of needs |
| Planning - develop structured implementation process description |
| Planning - thinking in advance about implementation |
| Tailoring strategies - Ensuring easy access to guideline content for end users (through guideline database  and patient information) |
| patient information stimulates implementation |
| Engaging - patient empowerment via patient-centered guideline information |
| Engaging - implementation support - communication advisor advises implementation team |
| Reflecting & evaluating – audit & feedback supports implementation |
| Individuals domain |
| Implementation leads x motivation - dedicated project leader needed |
| Innovation deliverers x need - demand for guideline |
| Innovation deliverers x capability - train implementation science practitioners to improve expertise |
| Innovation deliverers x motivation - intrinsic motivation/passion/drive to deliver good care |
| Inner setting domain |
| Structural characteristics - information technology infrastructure alignment |
| Structural characteristics – healthcare professionals challenge each other at network meetings |
| Relational connections - short lines between healthcare professionals, managers, ICT |
| Culture – healthcare facility and professionals are learning centered |
| Culture – culture of acting according to the guideline ingrained within profession |
| Available resources - funding available to implement guideline |
| Access to knowledge & information - implementation agenda raises awareness for the need for change |
| Outer setting domain |
| Local attitudes - implementation momentum/attention |
| Implementation named in quality policy vision |
| Organizations feel increased urgency |
| Local conditions - resources to guidelines because of proven added value |
| Shared beliefs that care quality + accessibility is important |
| Partnerships & connections - bond of trust between guideline organizations |
| Partnerships & connections: guideline organization’s alignment/agreements are basis for implementation  execution |
| Cooperation guideline organizations |
| Passende zorg/IZA |
| Landelijke transmurale afspraken |
| Contract health insurer and NVZ |
| ZE&GG efforts |
| Partnerships & connections - alignment between guideline sources |
| Policies & laws- healthcare professional has to justify actions to medical board |
| Financing - financial compensation in return for implementation action plan |
| Financing – guideline financiers impose different requirements regarding implementation |
| External pressure – IGJ presses implementation |
| Innovation domain |
| Innovation source - guidelines developed/endorsed by healthcare professionals and scientific  organizations |
| Scientific associations support implementation agenda |
| Innovation source - guideline source good reputation improves acceptability among users |
| Innovation evidence-base – guideline’s (perceived) credibility and evidence-base |
| Innovation complexity - Guideline recommendations that are concrete, practical, and straightforward,  without unnecessary complexity |
| Concrete formulation recommendations |
| Guidelines perceived relevance to practice |
| Innovation design - modular maintenance saves more energy for implementation + easier dissemination |
| Innovation design - recurring fixed guideline publication moment |
| Innovation design - guideline described from patient perspective |
