# Supplementary material for: Nationwide guideline implementation: a qualitative study of barriers and facilitators from the perspective of guideline organizations
Source: BMC Health Serv Res. 2025 Jan 27;25:150. doi: 10.1186/s12913-025-12270-2 (PMC11771117; doi:10.1186/s12913-025-12270-2)
Supplement: Supplementary file 4 — Supplementary Material 4: Implementation barriers and facilitators identified by representatives of guideline organizations, classified according to the updated CFIR framework. [file 12913_2025_12270_MOESM4_ESM.docx]

Supplementary material 4. Implementation barriers and facilitators identified by representatives of guideline organizations, classified according to the updated CFIR framework

| **Categories of implementation determinants (classified by the updated CFIR)** | **Barrier** | **Exemplary quote corresponding to the barrier** | **Facilitator** | **Exemplary quote corresponding to the facilitator** |
| --- | --- | --- | --- | --- |
| **Innovation domain** | | | | |
| **Innovation source** | Guideline, guideline committee and/or guideline developer predominantly reflect an (academic) professional/medical perspective, giving less consideration to other interests, stakeholder perspectives and contexts (e.g. patient or general hospital perspective) | ‘... Good care is now primarily described from a certain angle, so from the [viewpoint of the] medical specialist ... And we believe that good care should be much more described from the condition or from the patient's perspective and that you should consider the entire spectrum.’ – Representative 26, governmental agency | Guidelines developed by peers and endorsed by scientific organizations foster consensus and support for their use among healthcare professionals | ‘Those medical guidelines are naturally developed by medical specialists. We support them in that, so they are kind of owners of those guidelines and that naturally aids with implementation. It's not like we've made them and say: ‘you have to do it this way’. So in that sense, you could consider it as a kind of implementation strategy that is underlying there.’ – Representative 10, guideline developer |
|  |  |  | Acceptance of the guideline is influenced by the favorable reputation that scientific/professional organizations maintain among their associated healthcare professionals | ‘If you have a good reputation in that regard, parties think: 'hey, but if it's facilitated by [guideline organization], then it's beneficial for us.' Or: 'they understand us, or those kind of matters.' That also helps a lot, of course.’ – Representative 29, guideline developer |
| **Innovation evidence-base** | Guidelines quickly become outdated due to the constant influx of new evidence and the lengthy development process | ‘That such a guideline may already be outdated by the time it is used, because it contains knowledge from 3 or 4 years ago. At the time of authorization, it can actually be revised again.’ – Representative 23, governmental agency | Guideline’s (perceived) credibility and evidence-base | ‘The guidelines that implement most easily are simply the ones where the evidence is very clear. Where it's just very clear: that's the effect, that's better. Then you don't even need to create a guideline, so to speak. If the outcome is clear, then there is no problem at all in implementing the guideline. None at all.’ – Representative 31, guideline developer |
|  | Insufficient conclusive evidence, resulting in weak guideline recommendations | ‘... Then the risk is that they find guidelines too abstract and unspecific. That they say: ‘it says: consider doing this or that. I just want to know: what should I do?’ Sometimes they prefer more of a protocol than a guideline.’ – Representative 4, guideline developer |  |  |
| **Innovation complexity** | Multidisciplinary guidelines are harder to develop and implement due to the involvement of multiple stakeholders with different interests | ‘Now there's, for example, a guideline ... About physical fitness in oncological patients. That's obviously a highly multidisciplinary subject. But that also makes it very challenging. In that workgroup, they did indeed encounter the issue of: how do you implement that? – Representative 18, guideline developer | Guideline recommendations that are concrete, practical, and straightforward, without unnecessary complexity | ‘We receive a lot of criticism, including about how [guidelines] have become so complicated. So I find it very important that we put more effort into making things easier to apply. The easier it is, the smaller the chance of errors. And it could involve improving the presentation, or improving the format, like a card or a simple summary. But it could also be in the nature of the advice you give. If you give very complex advice, the risk of errors increases.’ – Representative 2, guideline developer |
| **Innovation design** | Guidelines are extensive documents, difficult to understand for some end users | ‘...That guideline on informal caregiving, for example, simply didn't resonate. It just didn't come across well, and that was largely due to how lengthy and complex it was.’ – Representative 4, guideline developer | Modular updating and structuring of guidelines save the guideline committee energy/time which they can re-allocate towards implementation efforts, enable faster incorporation of new insights, and make the recommendations more manageable to disseminate/implement | ‘That's why it's good that modular maintenance exists. ... Yes, because when you think in modules, you're no longer dealing with writing a 600-page guideline first ... Whereas if it's in smaller steps, then you're indeed making good progress. You've worked on it together for about a year, and it results in something of which you think: we can work with that. Then you can allocate the energy you’ve saved much more towards those other steps. So I think that's super important.’ – Representative 32, guideline developer |
|  | Guideline recommendations are formulated vaguely, as guideline committees are cautious about formulating strong recommendations, to preserve professional autonomy and avoid being held accountable by peers | ‘… You notice that there can sometimes be hesitancy in writing recommendations because they think, ‘well, that means we're now imposing on our peers to do a certain thing. We'll be held accountable for it.’ So you notice that sometimes it's also challenging when formulating recommendations. They may want to remain a bit vague to maintain freedom.’ – Representative 10, guideline developer | Recurring fixed guideline publication moment | ‘You know exactly when they are released; it's always at the September conference. So, they have a very powerful implementation tool and structure that is very reliable.’ – Representative 33, guideline developer |
|  |  |  | Guideline described from patient perspective | ‘What I do find positive about our quality standards, care standards, and generic modules is that they are written from the patient journey perspective. Because by putting that at the center, you create a kind of 'we are all going to work on this together' feeling. ... This also somewhat shifts the focus away from the professionals themselves, perhaps encouraging them to think, you know, what is important for that patient? ... By framing and labeling them like this, it's also something like, ‘oh yes, this is what we, together with patients, have come up with as important for the patient’. Instead of, ‘what do I need to do?’ And of course, that latter part is in there as well, but it's the framing that makes you think and act. I think that really helps.’ – Representative 29, guideline developer |
| **Outer setting domain** | | | | |
| **Local attitudes** | Belief that implementation occurs swiftly/automatically once a guideline is published and requires minimal resources | ‘Additionally, there is a need for greater awareness in the field that implementation does not happen automatically. I think that if you ask an average physician: ‘how do you implement or how does your department implement new knowledge into practice?’, they would say: ‘We give a presentation, and then it's implemented’. So, there is still much progress to be made in this regard.’ – Representative 1, national organization | Increased implementation momentum/urgency/more attention to implementation | ‘And that, that's really going to change now, because I think there is a need from scientific organizations to do something about it. There is much more attention for it now. Just like we thought a while ago, we should do something about it, others are thinking the same now.’ – Representative 32, guideline developer |
| **Local conditions** | Due to the increasing demand for care and limited care provision resources, concessions must be made regarding quality, accessibility, and affordability, which means that not all guideline recommendations can be implemented | ‘But due to the increasing demand for care and limited resources to provide that care, many concessions will have to be made regarding quality. And regarding accessibility and affordability.’ – Representative 35, governmental agency | Due to the increasing demand for care and limited care provision resources, more attention is given to implementing appropriate care described in guidelines | ‘We face challenges in healthcare on all fronts, including accessibility, staffing, and affordability. When it comes to making critical decisions about where to allocate our limited workforce, we prefer to focus on care that we know adds value for the patient.’ – Representative 1, national organization |
| **Partnerships & connections** | Guideline organizations do not have a clear view of the similarities and collaboration opportunities regarding each other's quality initiatives | ‘If one club has improvement reports, the other has improvement goals, and they both relate to something cardiological, yes, then you should merge them into one narrative. … And having the right conversation about it, we haven't quite gotten there yet. We simply haven't fully clarified where the intersection lies.’ – Representative 35, governmental agency | The bond of trust between specific (representatives of) guideline organizations strengthens implementation efforts | ‘Because we collaborate quite intensively, a bond of trust has developed among us, reflecting and shaping our shared commitment.’ – Representative 32, guideline developer |
|  | Central and local stakeholders (e.g. insurers, healthcare professionals, healthcare facilities’ board of directors) do not collaborate effectively due to mutual distrust | ‘That's a bit how they look at each other. Yeah, and if I trust you, then I want to help you get that done. ... Collaborating isn't saying, 'you have to do that'. Collaborating is thinking together, ‘what do we have, what needs to happen, and what can I do?’ And we're really not there yet, are we? ... That lack of collaboration, lack of trust, it's in all parties.’ – Representative 32, guideline developer | Critical sources/ websites containing guideline information (e.g. Thuisarts and websites of scientific/ professional organizations) are updated and aligned | ‘Yes, and besides that, I also think [a facilitating factor] is the reference to other guidelines, and guidelines also refer back to us.’ – Representative 18, guideline developer |
|  | Guideline organizations do not use all collaborative opportunities to inform stakeholders about guidelines | ‘And I think we could do even more in informing healthcare facilities, because we do have a network, but we don't have all hospitals exactly in sight, knowing what they all do or don't do. You expect your stakeholders to inform each other and their colleagues elsewhere in the country. But, that often doesn't happen. As [guideline organization] we do have the policy, which I actually don't fully agree with, that we don't send our reports to the hospitals’ Boards of Directors. Then you can't assume that every hospital is aware that something needs to be done.’ – Representative 6, governmental agency | Formal collaborative agreements between guideline organizations (e.g., ZE&GG program) ensure a collective commitment to implementation and provide opportunities to more easily reach multiple stakeholders | ‘Know that there is increasingly more commitment at this moment among all relevant parties essential for translating the developed knowledge into implementation. And this is not the responsibility of just one party. It involves all parties. And that's what the ZE&GG program is currently striving to achieve: to emphasize that everyone has a role to play in this endeavor.’ – Representative 14, governmental agency |
|  | Not all (guideline) organizations agree with certain guideline recommendations | ‘So the scientific organizations may believe that substantive improvements are necessary. However, the hospitals, together with their medical staff, must implement it, and if the Dutch Hospital Association (NVZ) has not agreed to it, then it is not so easy to proceed with it.’ – Representative 6, governmental agency |  |  |
| **Policies & laws** | Regulations (e.g. volume standards, rules about fair cooperation between healthcare facilities from the Authority for Consumers and Markets (ACM)) hinder implementation | ‘The fact that there are volume standards. It can be hugely inhibiting... It may be that by applying [the guideline], you fall below your volume standards. And then you can no longer provide care, and then care needs to be relocated elsewhere or you need to collaborate... And then someone says, ‘well, let's check this with the Authority for Consumers and Markets. Oh, no, that's a form of collaboration that is not allowed’... So you may very well have conflicting interests, regulatory issues.’ – Representative 32, guideline developer | Healthcare professional must justify themselves to a disciplinary or incident review board due to an incident/complaint and undergo assessment to determine if they have worked in accordance with the professional standard | ‘A negative situation is when you experience a complication and you have to appear before the disciplinary board or incident review board because you need to justify yourself. Yes, those who have experienced it once will definitely read the guidelines the next time. But that's only one in so many cases. You can't wait for everyone to close the stable door after the horse has bolted.’ – Representative 17, guideline developer |
|  | Conflicts between different guidelines/protocols on the same topic | ‘If you compare standards now, you can also see the discrepancies. So as a healthcare professional, you have to figure out what to do with it: 'oh, this [standard] prescribes this and that [standard] prescribes that. What on earth am I supposed to do with that?'’ – Representative 26, governmental agency |  |  |
| **Financing** | Many guideline organizations lack standard budgets for implementation and instead depend on supplementary external funding | ‘But that often relies on additional subsidy funds, so it's not standardly included. … There are some tools and projects where, for example, an extra workgroup focuses on implementation, but all of those are things that have been specifically requested as extras.’ – Representative 10, guideline developer | Various funders of guidelines require guideline developers to meet implementation criteria as a prerequisite for funding the development of guidelines | ‘That varies, you know, because with [guideline organization A] it's really a side issue, and with [guideline organization B] it's also somewhat important, but if it's funded by [guideline organization C], then implementation becomes more significant, and they often have to conduct pilot implementations.’ – Representative 27, national organization |
|  | The connection between quality of care and care reimbursement/financial incentives is weak and challenging to establish in the current healthcare system | ‘You actually want to move away from incentives, money, and focus more on quality, so efforts are being made on various fronts to achieve that. However, it doesn't change the fact that market dynamics inherently have incentives that make money important, I think.’ – Representative 23, governmental agency | Healthcare facilities receive financial compensation in exchange for creating an implementation action plan | ‘So, there's an obligation to make efforts. ... So, each hospital must create an implementation plan for the Implementation Agenda, and in return, they receive a 1.62% increase in all prices to compensate for wage increases and similar expenses. That's a bit of a motivator: you get a bit more money from the healthcare insurers, however, you must make a plan for ZE&GG every year at the very least. That's a very strong promoting factor.’ – Representative 1, national organization |
|  | Fear of reduced revenue due to the de-implementation or relocation of certain care practices as recommended in the guideline | ‘Yes, because that often involves livelihoods, right? ... It means more people going to the hospital and fewer being treated in primary care. That comes at the expense of the wallet of those in primary care. And people naturally don't like that. Well, those kinds of things all come into play there.’ – Representative 31, guideline developer |  |  |
|  | Implementation projects/ programs and their funding are temporary and lack structural updates, leading to unsustainable implementation or continued use of outdated guidelines/ implementation tools | ‘... But that's also linked to a guideline from 2011 or 2013, that program. And then the funding stops, but there's no budget left to revise or revisit it. … But it's also good to de-implement choosing wisely recommendations if they're no longer valid, or to review them.’ – Representative 33, guideline developer |  |  |
| **External pressure** | Guideline organizations have insufficient mandate/power to push implementation, compared to significant healthcare professional autonomy | ‘I think that might also be one of the biggest obstacles we face as [guideline organization], that we provide a lot of advice, deliver reports, facilitate, but actually, we can't do much when it comes to implementation. You see, if it doesn't happen, we don't really have leverage. That sounds very negative, but yeah, then it kind of stops there. So we are mainly facilitative and directional.’ – Representative 23, governmental agency | Health and Youth Care Inspectorate (IGJ) can use its authority/power to push implementation in healthcare facilities | ‘Only, [the IGJ] had enforcement power. On the other hand, [the IGJ] also relied on what the doctors had decided because the Inspectorate doesn't come up with anything new. It doesn't say: ‘you must do this or that’. But if the doctors say: ‘this is the best practice and it's not happening’, then the Inspectorate can enforce it at some point: 'Sir or Madam, why are you doing something different? Because we've all agreed on this’. That was a very good collaboration, keeping each other informed of what we're doing ... So we moved forward together. But it does indicate: it's a strong driver when the IGJ says something must be done.’ – Representative 17, guideline developer |
|  | Priority towards developing/revising guidelines rather than disseminating/implementing them | ‘A barrier is the competition between the time spent on revising the guideline versus the time spent on dissemination and implementation.’ – Representative 15, guideline developer |  |  |
| ***Available capacity/ resources^a^*** | Guideline organizations lack the capacity/resources to handle the multitude of (quality) tasks, including implementation of guidelines, and therefore have to determine which quality initiatives to prioritize | ‘I think what's very important is: there are no resources available. A lot is being demanded from scientific organizations, also by the government. Through agreements and through guidelines, and yeah, it's not possible to do everything. ... So it's really just resources and time.’ – Representative 33, guideline developer | *No facilitators were mentioned in this category.* | |
| **Inner setting domain** | | | | |
| **Structural characteristics** | *No barriers were mentioned in this category.* | | Aligning information technology infrastructure with the guideline | ‘What I believe works even better, and that's currently happening with the fracture prevention project: there's a new guideline. Now, an [electronic health record software] module is also being adapted and implemented. ... So if you present it ready-made, such a system they can easily adopt, then it does happen.’ – Representative 6, governmental agency |
|  |  |  | Structural attention to guidelines in healthcare professional practice meetings (e.g. handovers, multidisciplinary consultations) | ‘At the moment, the most you can do is hold each other accountable. For example, large interdisciplinary meetings or handover moments can help. Like the morning handover where admitted patients are discussed, or during a case discussion where someone says, hold on, don't we have guidelines for this? It says this and that.' So, holding each other accountable in that way. Those are the tools that work best.’ – Representative 17, guideline developer |
| **Relational connections** | *No barriers were mentioned in this category.* | | Healthcare facilities with short communication lines between healthcare professionals, support functions (IT department), and management adapt to guidelines more quickly | ‘In a clinic, when they say: we need to adjust the process, they adapt the entire process, and it's usually done quite quickly. Because communication lines are very short in clinics, things can happen very quickly. The medical director is often also a physician and a manager, and they often still see patients. So, if they understand that changes need to be made, they adjust their processes, and the entire clinic follows suit all at once.’ – Representative 7, national organization |
| **Communications** | Open communication across hierarchical structures (e.g. work experience, nurse-physician relationship) about changing practice is perceived as challenging | ‘Then a nurse, fresh from training, arrives. They come with new knowledge to a department, and a nurse who has been working there for many years might say, ‘we simply don't do this here’, for example, or ‘we do this, but in a different way’. And we see that for instance younger nurses find it very challenging to have these kinds of conversations with their colleagues.’ – Representative 12, national organization | *No facilitators were mentioned in this category.* | |
| **Culture** | *No barriers were mentioned in this category.* | | Healthcare facility and healthcare professionals are learning-centered | ‘As a physician, you tend to do things automatically because it's how you've always done them. So, it's important to stay updated on the latest insights, to value the innovation of your field, to prioritize lifelong learning, to maintain interest in both the individual patient and the broader population. It's not just about performing actions that are deemed correct, but also considering the impact of those actions on the patient and beyond the facility. All these aspects play a role in implementation. It's not just about writing down and saying: 'This is how we'll do it now.' It's about taking action, changing behaviors, and learning new things.’ – Representative 27, national organization |
|  |  |  | Culture of adhering to guidelines ingrained within the profession | ‘I don't have specific numbers, but I believe we have a relatively high level of guideline implementation. I can't provide specific success stories off the top of my head, but I think within [specialist profession], and also from what I gather from medical training, there's a strong adherence to the guidelines. Deviation is always allowed, but the guidelines have been quite well integrated within the professional group.’ – Representative 2, guideline developer |
| **Relative priority** | Healthcare facilities need to determine which quality initiatives (e.g. programs, guidelines) to prioritize and implement, given the abundance of initiatives, limited capacity/funding and the compatibility of the requested organizational changes | ‘But it would be good if [quality initiatives] were more connected to each other, because you see hospitals being overwhelmed with programs ... Laws, regulations, and guidelines are things you really have to comply with, and external accountability of quality figures, for example... But we simply don't have time to do all that.’ – Representative 5, national organization | *No facilitators were mentioned in this category.* | |
| **Available resources** | *No barriers were mentioned in this category.* | | Healthcare facility has allocated funding to enable healthcare professionals to dedicate time to implementation | ‘The nurses who are working on it, also receive time for it from the hospital. So they get a few hours per week to dedicate to [program]. Because ultimately, it should also save time for the nurse, as they are omitting various tasks that do not add value for the patient.’ – Representative 1, national organization |
| **Access to knowledge & information** | *No barriers were mentioned in this category.* | | Implementation Agenda raises awareness within healthcare facilities about the need for implementation and their responsibility in the process | ‘The health insurers and hospitals have made an agreement that they will have implemented a certain percentage of the Implementation Agenda in hospitals within a certain timeframe. This has made hospitals aware of their responsibility in implementation.’ – Representative 32, guideline developer |
| **Individuals domain (roles subdomain / characteristics subdomain)^b^** | | | | |
| **Implementation leads / motivation** | *No barriers were mentioned in this category.* | | Dedicated implementation leader | ‘But there must be passionate healthcare professionals who are willing to help other hospitals implement something. Because you also see that with implementation research, there really needs to be a project leader who needs to be freed up to implement something. ... So there must be a program leader or project leader who initiates things, as with ‘Choosing Wisely', and then you get things done.’ – Representative 6, governmental agency |
| **Implementation team members / capability** | Guideline committee insufficiently considers or lacks the necessary expertise to address the implementability of the guideline during its development process | ‘The success of implementation also depends on how well the guideline is constructed. Has implementability been adequately considered in that guideline? Is there sufficient capacity to make changes? Is the physician population ready to tackle things differently? Is there enough funding? Is the hospital willing to invest? So, those are all aspects that you actually need to discuss very thoroughly when [developing] a guideline. And I see that that is not always the case.’ – Representative 27, national organization | *No facilitators were mentioned in this category.* | |
|  | Guideline committee lacks adequate expertise in planning/executing implementation strategies | ‘But the people who sit on the guideline committee, they are a different type of people. They are not people who are engaged in actively moving things forward, but rather on researching or searching the literature. … But they are not the type of people who say, ‘Hey, why haven't you done that yet?’’ – Representative 17, guideline developer |  |  |
| **Implementation team members / opportunity** | Guideline committee has insufficient capacity/time to plan/execute implementation strategies | ‘There is substantial brainstorming about creating podcasts, webinars, micro learning quizzes, all these methods to get guidelines to the user. ... However, this often depends heavily on the commitment of guideline committee members. Whether such ideas actually materialize depends on factors like budget and time availability. But very often, it seems like voluntary work from the committee members to engage in these extra activities.’ – Representative 18, guideline developer | *No facilitators were mentioned in this category.* | |
| **Implementation team members / motivation** | Guideline committee lacks motivation to plan/execute implementation strategies | ‘Well, one thing is whether a committee member wants to do it. I think that's actually quite important. For example, if you look at creating educational material, if no one feels compelled to create or write it, well, then it doesn't happen. Because I'm not going to do it. I don't have the subject matter expertise.’ – Representative 15, guideline developer | *No facilitators were mentioned in this category.* | |
| **Innovation deliverers / need** | *No barriers were mentioned in this category.* | | There is a strong demand among healthcare professionals for a specific guideline (e.g. there is a high level of uncertainty) | ‘For example, that guideline on asthma and pregnancy is highly appreciated, I know. I think it's because everything wasn't quite well-known yet, and people were really searching for some certainty and something to rely on. So, there was just a great desire for such a guideline, you can really feel it.’ – Representative 34, guideline developer |
| **Innovation deliverers / capability** | Certain medical professional groups are not yet accustomed to using guidelines | ‘... I think that for example, for medical specialists, it is very natural that there is a guideline and they also know what is in that guideline and act accordingly. However, I think that's not necessarily the case for our professional group. I think we are becoming increasingly aware of this and figuring out how we are going to approach it. We can develop beautiful things, but we also need to ensure that this awareness is established in the first place.’ – Representative 3, guideline developer | Train implementation science practitioners to improve implementation expertise | ‘We have developed a 1.5-year training program for implementation coaches. Bachelor-level nurses have completed this program, where they learn the knowledge and skills needed to implement a guideline within their organizations.’ – Representative 3, guideline developer |
|  | Healthcare professionals are hesitant to (de-)implement out of fear of causing harm to patients | ‘Everyone is more inclined towards the paradigm of always doing something because it might help, rather than doing nothing, because then you don't know. ... And perhaps then I have to face the disciplinary judge, and then all of the Netherlands will come down on me.’ – Representative 25, health insurer |  |  |
|  | Healthcare professionals have insufficient implementation expertise | ‘Yes, especially the expertise in implementing within the field. At the moment, that’s a very important component, which we currently see is insufficient.’ – Representative 14, governmental agency |  |  |
|  | Healthcare professional’s lack of confidence/ knowledge/skills to execute guideline | ‘So, that also reveals another barrier, namely skills, and also sometimes the lack of self-confidence. That a guideline is just very high in information and complex to understand.’ – Representative 4, guideline developer |  |  |
|  | Healthcare professionals’ fear of going against patients’ expectations/wishes | ‘But when a patient makes a request to you, it's very difficult to say no.’ – Representative 25, health insurer |  |  |
| **Innovation deliverers / opportunity** | Healthcare professionals struggle with keeping track of and implementing the multitude of guidelines due to limited capacity and time constraints | ‘I think there are just a lot of guidelines, and I wonder how anyone could possibly have all of them memorized in intricate detail. It makes me wonder, guys: it's simply not humanly possible to adhere to all of them.’ – Representative 26, governmental agency | *No facilitators were mentioned in this category.* | |
| **Innovation deliverers / motivation** | Healthcare professionals experience guideline fatigue | ‘There is also something called change fatigue, so that something new keeps coming up and people are fed up with it. ... There are simply limits to what people can handle.’ – Representative 11, national organization | Healthcare professionals are intrinsically motivated to improve and deliver good care | ‘... Nurses feel the responsibility very strongly. 'If I don't do it right, I put my patient in danger, so I want to know exactly what I need to do.' That's really beneficial because it means they're also willing to look at that guideline, or at least willing to listen when we say, 'listen, with this or that topic, things aren't going quite smoothly. So we've developed a guideline.' They feel that necessity.’ – Representative 4, guideline developer |
|  | Healthcare professionals cling to old habits and routines | ‘You are still open to learning new things now, but if you've been at it for 30 years already, doing it for 30 years based on a certain belief and with a certain experience. Yes, then it's more difficult to change your behavior than if you're still open to learning.’ – Representative 25, health insurer |  |  |
|  | Healthcare professionals experience cognitive dissonance between their past/current practices and the guideline-recommended practices | ‘Cognitive dissonance. ... Before you overcome that, it really requires you to be open to the question: I used to do it right, but maybe today things are different and better. ... That's quite an insight to have, and I imagine it's not really pleasant." – Representative 17, guideline developer |  |  |
|  | Healthcare professionals perceive guidelines as restricting their professional autonomy | ‘Another inhibiting factor is that many professionals believe that protocols, guidelines, and standards limit their [practice] freedom. They think that this prevents them from delivering personalized care effectively. … They consider it important to deliver good and personalized care based on the patient's needs, viewing guidelines and standards as constraints.’ – Representative 29, guideline developer |  |  |
| **Implementation process domain** | | | | |
| **Assessing needs** | *No barriers were mentioned in this category.* | | Through close engagement with stakeholders, guideline organizations can better understand their implementation needs and effectively facilitate them | ‘So I think it's especially the very close contact with the intended target audience. In that regard, I believe we deviate somewhat from what other policy organizations do. Instead of sitting in an ivory tower, coming up with what the field needs, we genuinely try to gather from the field itself what they need and facilitate them in that.’ – Representative 1, national organization |
| **Planning** | Guideline implementation plan is very concise, not concrete and/or copied from a previous guideline | ‘That's really an issue that needs much more attention. You see all the umbrella organizations throwing themselves with all their verve, energy, and resources into developing guidelines. And then, in essence, the implementation is just a standard A4 sheet, where they, so to speak, put the guideline title on top, fill in a few empty text fields, and hang it in the guideline database. And then hope that the doctor finds that guideline and applies it.’ – Representative 27, national organization | Creating a detailed step-by-step plan for the implementation process | ‘We described the patient information process very well, detailing how we would proceed. We did this for summary cards. And for other things. Like news items. So, we've created several good process descriptions. I find that beneficial.’ – Representative 15, guideline developer |
|  | No adequate attention/follow-up/action to guideline implementation plan/pilot implementation | ‘There should be an implementation plan. That is delivered within the guideline, but actually, very little is done in executing the plan, and that's really the step that still needs to be taken, for quite some time now.’ – Representative 21, guideline developer | Addressing implementation and engaging stakeholders early on, already in the guideline development process | ‘Thinking ahead about how you can potentially map things out with benchmarking data, which we know can be very useful for implementation. If you're already thinking about this in the way you develop the guideline. And involving relevant stakeholders in a timely manner. … So that you can address any obstacles upfront instead of trying to patch things up afterwards. – Representative 1, national organization |
|  | Implementation is not addressed throughout the process, but only in the last phase | ‘It's also somewhat the case that with a guideline, implementation is the final piece, so it's the last thing people do. 'Oh, you also need to make an implementation plan' and then there's relatively little attention given to it.’ – Representative 32, guideline developer |  |  |
| **Tailoring strategies** | Guidelines are published in different guideline databases which makes it less user-friendly and creates uncertainty about guideline currency and quality | ‘And we see that a number of organizations [lists organizations] actually do not use the Registry. And that leads to contamination. This results in outdated quality standards being included in the Registry of the National Health Care Institute. This leads to a lack of transparency. That was a goal in itself of the Registry, that it is transparent what is considered good care. That is not the case when every professional group maintains its own guidelines database.’ – Representative 26, governmental agency | Ensuring easy access to guideline content for end users (through guideline database and patient information) | ‘Well, a significant step has already been taken with the guideline database, ensuring it's located in a central place and not kept behind restricted access websites.’ – Representative 9, guideline developer |
|  | Implementation strategies are not tailored to guideline end users | ‘Furthermore, these guidelines are published in the guideline database of the Dutch Association of Medical Specialists. ... I just notice that they are less easily accessible for our target audience there, as it doesn't entirely align with the information needs system of [medical specialist], and perhaps other medical specialists as well.’ – Representative 33, guideline developer |  |  |
| **Engaging** | Forgotten/late engagement of stakeholders in the implementation planning/execution | ‘Yes, but then it's important that [guideline organization] is involved as early as possible, to ensure that the entire document, along with all recommendations, isn't finalized prematurely, leading to the subsequent discovery that it’s not feasible. It's better to discuss that at the beginning of the process.’ – Representative 5, national organization | Empowering patients through the development of guideline information specifically for patients | ‘But the patient in the waiting room says, ‘Hello. I've read that guideline, you know, because I received it from my patient association, which has translated it into text that we understand. Why haven’t you mentioned that treatment option?’ Yes, it can also be justified for the doctor to say, ‘you have a completely different diagnosis, but please explain.’ This also keeps the doctor sharp. – Representative 17, guideline developer |
|  |  |  | A communication advisor is involved and assists implementation | ‘Well, I find it helpful that a communications advisor is very closely involved with us. ... They sometimes see opportunities that we might not notice ourselves. Like, ‘Oh, here's a good opportunity for a news item’, and then you think, ‘Oh yeah, that's newsworthy too’. ... I find it helpful that she observes, reads, asks questions, gives advice; I find that beneficial.’ – Representative 15, guideline developer |
| **Doing** | No standardized guideline development and implementation process | ‘Then you should really take a moment to consider: how can we ensure that we streamline it as much as possible? So that it's easier for everyone to understand. All those different definitions of guideline types, the entire process, all those different entities involved. It should be more standardized.’ – Representative 5, national organization | *No facilitators were mentioned in this category.* | |
| **Reflecting & evaluating** | Insufficient good quality data on the success of implementation | ‘What we really miss in these kinds of discussions is data. How does it actually work in practice? How often do we go left? How often do we go right? How significant is the practice variation? What else do we need to achieve implementation? So, we're really missing data. We would really like to have that.’ – Representative 32, guideline developer | Audit & feedback benchmark information is useful to support implementation and evaluation | ‘Measuring certain outcomes allows you to say, ‘wait a moment, these outcomes could be better, what is the problem?’ And then you come back to the guideline: ‘oh yes, others do this differently and that's why it's better’. So, in addition to education, also evaluating what you have done. So, looking back, sometimes setting up dashboards to see how you are doing. Anyway, providing feedback helps.’ – Representative 17, guideline developer |
|  | Balancing the need for data collection for measuring implementation success against the perceived burden of administration is challenging | ‘In an ideal world, you would collect precise data for the specific topics on the Implementation Agenda. So literally, people would have to input how often they still do something. Because then you can uniformly extract from each system how people are doing with implementation. But, of course, that imposes a massive administrative burden, which is not desirable at all. Because ultimately, people should be able to provide care and not spend the whole day behind a computer.’ – Representative 1, national organization |  |  |

Implementation determinants are classified according to the updated CFIR [1]. Definitions and detailed descriptions of the updated CFIR concepts are presented in the additional files of Damschroder et al. (2022).

Quotes are translated from Dutch.

^a^Implementation determinants that did not fit in the original updated CFIR were classified and added in *italicized* text.

^b^In the individuals domain, we coded the relevant characteristics for each identified role, following the recommendation of Damschroder et al. (2022).

**Reference**

1. Damschroder, L.J., Reardon, C.M., Widerquist, M.A.O., et al. *The updated Consolidated Framework for Implementation Research based on user feedback.* Implement Sci, 2022. **17**(1): p. 75. DOI: 10.1186/s13012-022-01245-0.
